# Klotho is upregulated in human cardiomyopathy independently of circulating Klotho levels

**DOI:** 10.1038/s41598-018-26539-6

**Published:** 2018-05-30

**Authors:** G. Poelzl, S. K. Ghadge, M. Messner, B. Haubner, Ph. Wuertinger, A. Griesmacher, J. Doerler, C. Ensinger, H. Ulmer, M. M. Zaruba

**Affiliations:** 1 Department of Internal Medicine III, Cardiology and Angiology, Medical University of Innsbruck, Innsbruck, Austria; 2 Central Institute for Medical and Chemical Laboratory Diagnosis, Medical University of Innsbruck, Innsbruck, Austria; 3 Department of Pathology, Medical University of Innsbruck, Innsbruck, Austria; 4 Department of Medical Statistics, Informatics and Health Economics, Medical University of Innsbruck, Innsbruck, Austria

## Abstract

Klotho is an antiaging protein which exerts known cardioprotection. In kidney, trans-membrane Klotho acts as essential co-receptor of fibroblast growth factor 23 (FGF23). In the heart, soluble Klotho (sKlotho) protects from systolic dysfunction independently of FGF23. Here, we analyzed the association of FGF23 and sKlotho upon progression of chronic heart failure (CHF) and analyzed Klotho expression in human hearts. Serum levels of sKlotho and FGF23 were measured in 287 patients with cardiomyopathy (CMP). Tissue samples from CMP (n = 10) and healthy control hearts (n = 10) were analyzed for Klotho mRNA and protein expression. Individuals in the first FGF23 tertile were 4.1 times more likely of freedom from death, heart transplantation or assist device implantation compared to third tertile. No relationship was found between sKlotho and the combined endpoint. Instead, Klotho mRNA encoding the full-length form was upregulated in human CMP hearts. Immunoblotting confirmed upregulation of sKlotho associated with increased expression of proteases involved in cleavage of Klotho suggesting rather local effects of Klotho in the heart. Therefore, we conclude that in contrast to FGF23, serum sKlotho is not associated with disease severity or progression in CHF. Instead, Klotho is expressed and upregulated in diseased hearts, suggesting local paracrine effects.

## Introduction

Chronic heart failure (CHF) remains a major public health issue. Prevalence of CHF is constantly rising and despite the best current treatment, disease-related hospitalizations are frequent and outcome is poor, with a one-year mortality rate of 6% to 24%^1,2^. Hence, strong emphasis is put on the identification of new targets to better understand the complex pathophysiology of CHF and develop novel treatment strategies.

In clinical studies elevated levels of phosphatonin fibroblast growth factor 23 (FGF23), an endocrine hormone secreted from osteoblasts and osteocytes that regulates phosphate homeostasis, have been linked to cardiac remodeling and the advent and progression of CHF^3–8^. The classical effects of FGF23 in the kidney and parathyroid glands are mediated by its binding to FGF receptor-1 (FGFR-1) complexed to the co-receptor Klotho, thereby increasing the binding affinity of FGF23 for FGFR^9,10^. Activation of the membranous Klotho-FGFR co-receptor complex by FGF23 suppresses renal synthesis of 1,25 dihydroxyvitamin D and inhibits renal phosphate reabsorption^9,11,12^. In the heart FGF23 has direct hypertrophic effects on cardiomyocytes mediated via FGF receptor-dependent activation of the calcineurin-NFAT signaling pathway^5^. Klotho is a type 1 membrane protein that exerts known antiaging function and is actively involved in the prevention of arteriosclerosis^13–15^. Deficiency of Klotho is known to shorten lifespan and leads to a phenotype resembling human aging^13^, whereas overexpression of Klotho extends lifespan by 20–30%^15^. Klotho is predominantly produced in the kidney, and circulating levels of soluble Klotho (sKlotho) are reportedly decreased in chronic kidney disease (CKD)^16,17^. The extracellular domain of Klotho can be cleaved by the α-secretases ADAM10 and ADAM17 and released into the circulation^15,18^. A first cut close to the cell membrane (alpha-cut) results in a 130 kDa circulating protein, and a second cut (beta-cut) between the KL1 and KL2 domains produces two smaller proteins with molecular sizes of approximately 65 kDa^19^. Both the full-length and the shorter forms of soluble Klotho (sKlotho) are hormones, with distinct but overlapping effects. In addition, a secreted isoform (isoform 2) arising from alternative RNA splicing has been described, however, does not seem to be translated into a functional protein^19,20^. In rodents sKlotho exerts cardioprotection through downregulation of TRPC6 calcium channels^21^. Moreover, in a CKD mouse model decreased levels of circulating sKlotho were an important cause of uremic cardiomyopathy independently of FGF23^21,22^. In hemodialysis patients, higher sKlotho levels were associated with the absence of atrial fibrillation^23^. From these data it appears that sKlotho might oppose the detrimental effects of FGF23 on the heart. Interestingly, so far Klotho has not been detected in cardiomyocytes^5,9,13^.

Based on the data published on Klotho in cardiac remodeling, we hypothesized that sKlotho might be inversely related with disease severity and progression in CHF. Furthermore, we examined the role of Klotho in human specimens of cardiomyopathy (CMP) and non-failing hearts.

## Results

### Study Cohort

The study cohort of 287 patients with chronic heart failure due to non-ischemic CMP and reduced left ventricular ejection fraction (LV-EF) comprised idiopathic CMP (47%), virus-negative inflammatory CMP (33%), and various etiologies including hypertrophic CMP, cardiac amyloidosis, and cardiac sarcoidosis (20%).

### Cross-sectional relations between FGF23, sKlotho and heart failure severity

**Figure 1:**
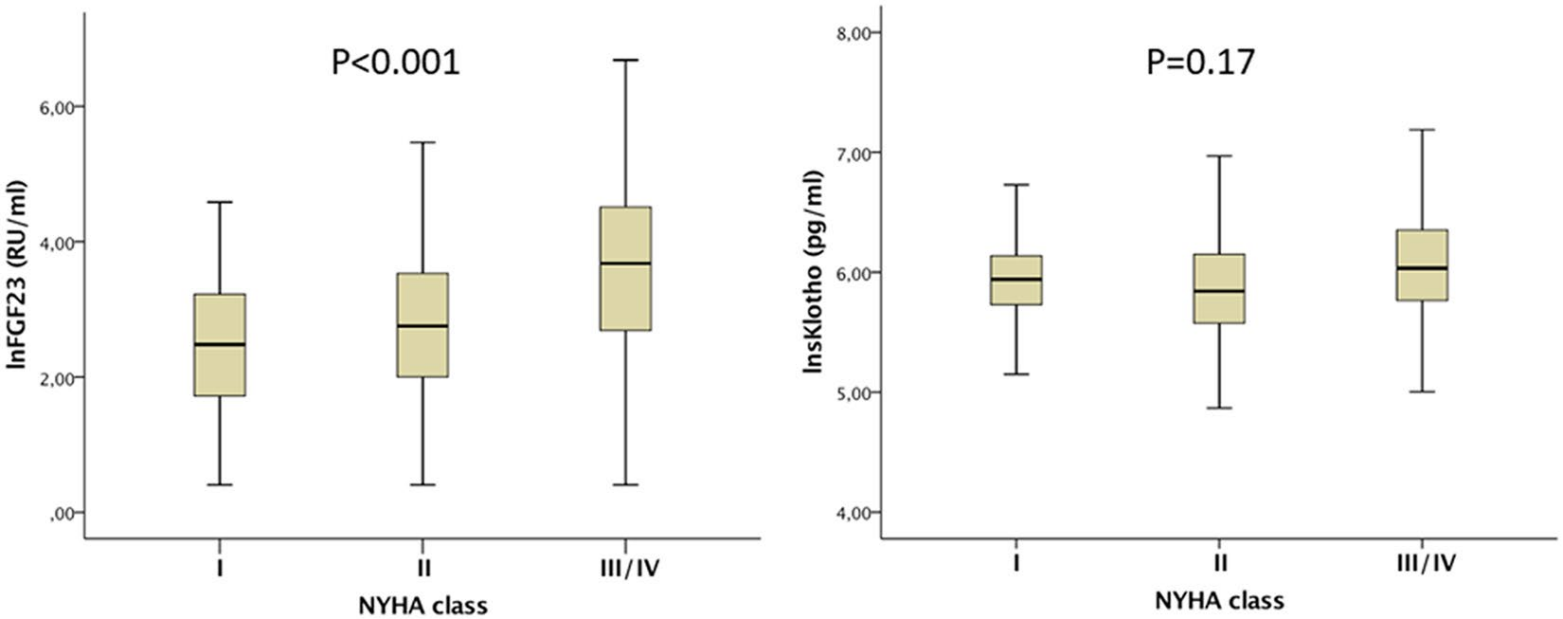
FGF23, but not sKlotho is related to heart failure severity. (**A**) Box-blot diagram showing median values of lnFGF23 related to NYHA functional class (p < 0.001 between the NYHA groups). (**B**) Box-blot diagram showing median values of lnsKlotho related to NYHA functional class.

Median levels of FGF23 and sKlotho in the plasma were 21.8 RU/ml (IQR 12.1–45.7) and 380 pg/ml (IQR 301–529), respectively. Baseline characteristics according to the median of FGF23 are shown in Table 1 and for sKlotho in Supplementary Table S2. High FGF23 levels (>20.17 RU/ml) were associated with significantly increased NT-proBNP, serum phosphate, parathormone, CVP, mean PAP, and PCWP levels, whereas 25(OH) vitamin D and CI were significantly decreased (Table 1). As shown in Fig. 1, a dose-response relationship was found between median FGF23 levels and increasing NYHA class (I: 16.5 RU/ml, II: 20 RU/ml, III/IV: 38.4 RU/ml; p < 0.001), but not for sKlotho (I: 380 pg/ml, II: 351 pg/ml, III/IV: 417 pg/ml; p = 0.17). Also, FGF23, but not sKlotho, correlated with NTproBNP (r = 0.307, p < 0.001 and r = −0.083, p = 0.176). No relation was found between FGF23 and sKlotho (r = 0.053, p = 0.371). FGF23 showed an inverse correlation with 25(OH)D (r = −186, p = 0.012), whereas sKlotho was positively correlated with 25(OH)D (r = 161, p = 0.037).

**Table 1:** Patient characteristics.

Variable	Total cohort		FGF23 (≤20.17 RU/ml)		FGF23 (>20.17 RU/ml)		p
	n = 287		n = 131		n = 156		
	Median or %	IQR	Median or %	IQR	Median or %	IQR	
**Demographic and clinical characteristics**							
Age (years)	48	38–58	46	38–55	50	38–62	0.108
Gender (male)	68%		67.5%		69.2%		0.797
LV-EF (%) Ventr	32	21–46	35	25–49	30	20–45	0.034
Heart rate (bpm)	71	61–83	69	60–81	73	63–85	0.031
Syst. BP (mmHg)	120	110–135	120	110–140	120	110–133	0.250
BMI	25.5	22.7–28.4	25.9	23.3–28.6	25.2	22.4–28.2	0.180
NYHA Class							<0.001
NYHA Class I	24.1%		34.9%		16%		
NYHA Class II	44.4%		46.8%		35.9%		
NYHA Class III/IV	23.5%		18.5%		48.1%		
Duration of heart failure (months)	2	1–8	2	0.5–8	2	1–8	0.304
Hypertension	42%		37.3%		46.2%		0.143
A-Fib	11%		7.9%		12.8%		0.178
**Laboratory testing (serum)**							
sKlotho (pg/ml)	380	302–529	378	289–495	386	311–530	0.438
NT-proBNP (ng/l)	1191	437–3147	607	167–1497	2042	896–3897	<0.001
eGFR (ml/min/1.73 m^2^)	75	61–92	82	69–98	69	55–83	<0.001 1
P_i_ (mg/dl)	3.4	3.0–3.8	3.3	2.9–3.6	3.5	3.1–3.9	0.015
PTH (ng/l) 173	33.6	25.7–49.6	31.7	23.6–41.9	38.4	28.3–56.5	0.005
25(OH)D, nmol/l 181	44	27.9–65.5	50.6	33.7–69.6	39.2.0	24.2–59.6	0.019
**Hemodynamics**							
CVP, mmHg	9	6–13	9	6–11	10	7–14	0.001
Mean PAP, mmHg	27	19–34	22	17–32	29	22–35	<0.001
PCWP, mmHg	17	11–25	13	10–22	19	13–27	<0.001
CI, l/min/qm	2.0	1,7–2.5	2.3	1.9–2.7	1.9	1.6–2.3	<0.001
**Medication**							
ACE inhibitor/ARB	78.4%		77.0%		79.5%		0.459
Beta blocker	76.6%		73.8%		78.8%		0.267
MRA	37.9%		27.8%		46.2%		<0.001
Diuretics	56.4%		33.7%		73.1%		<0.001
Cardiac glycosides	5.0%		3.2%		6.4%		0.287

Data from 282 patients are reported as median (interquartile range) or number (percentage) related to low and high FGF23 plasma levels.

LV-EF, left ventricular ejection fraction; Syst. BP, systolic blood pressure; BMI, Body Mass Index; A-Fib, atrial fibrillation; NT-proBNP, N-terminal pro-B-type natriuretic peptide; eGFR, estimated glomerular filtration rate; PTH, parathormone; Pi, serum phosphate; 25(OH)D, 25-hydroxyvitamin D; CVP, central venous pressure; mean PAP, mean pulmonary artery pressure; PCWP, pulmonary capillary wedge pressure; CI, cardiac index; ACE inhibitor/ARB, angiotensin-converting enzyme inhibitor/angiotensin receptor blocker; MRA, mineralocorticoid receptor antagonist.

### FGF23 but not sKlotho predicts progression and outcomes in heart failure

Complete follow-up information was available in 283 (98.6%) patients. Thirty-seven (13.1%) patients reached the combined endpoint of death, heart transplantation or assist device implantation (death in 28, heart transplantation in 7, and VAD in 2 patients) within a median follow-up period of 634 [296–1073] days. In these patients’ serum levels of FGF23 (47.7 [17–100.6] vs 20.9 [12–39.6]; p = 0.004) were higher as opposed to lower serum levels of sKlotho (346 [257–420] vs (396 [309–552]; p = 0.034).

**Figure 2:**
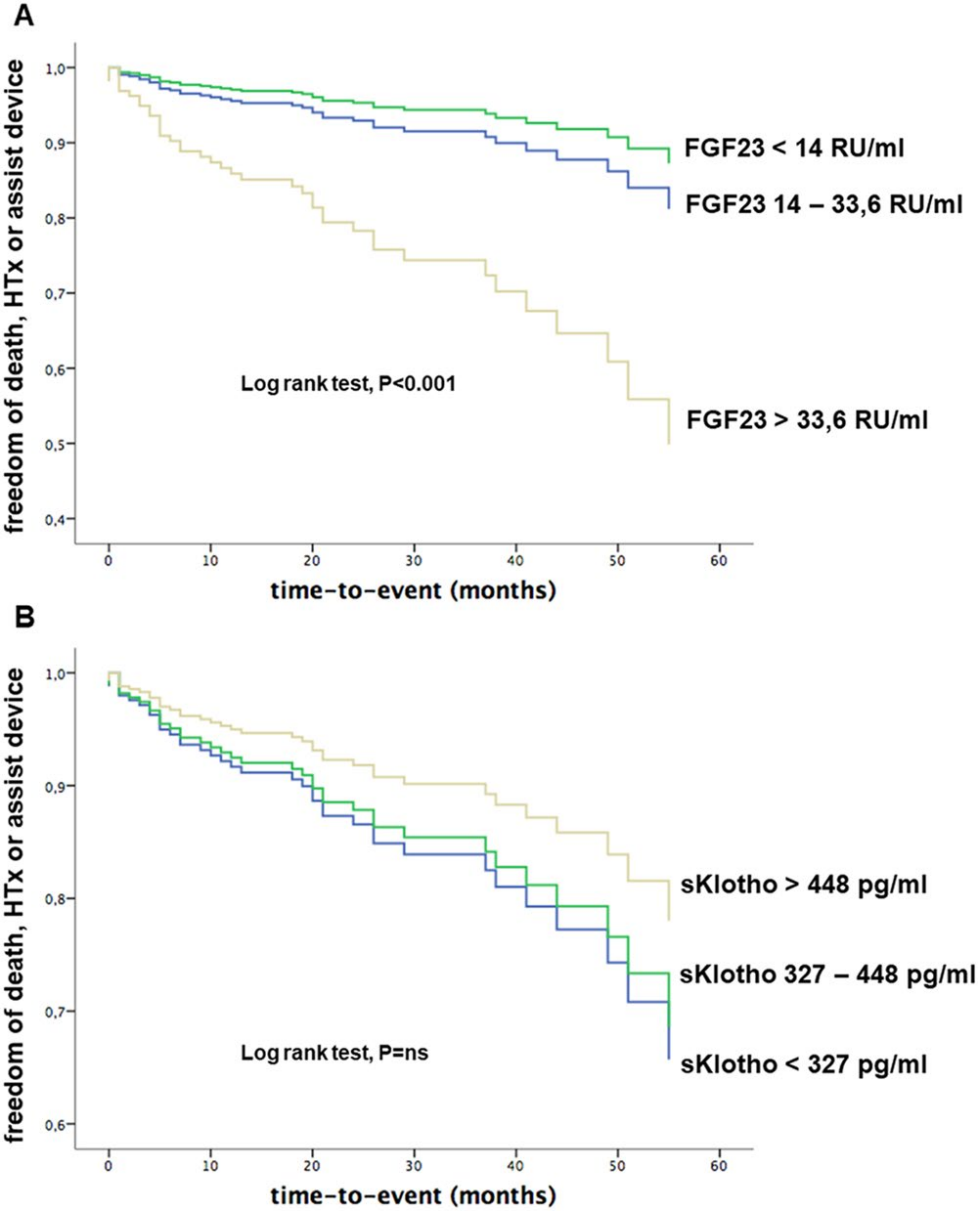
FGF23 and sKlotho in heart failure outcomes. Kaplan-Meier plots of FGF23 (**A**) and sKlotho (**B**) in tertiles each showing the combined endpoint comprising of freedom from death, heart transplantation (HTx) and assist device implantation. Log rank test revealed significantly differences between FGF23 tertiles P < 0.001, whereas sKlotho groups did not significantly differ from each other.

In Cox regression analysis adjusted for age and sex, FGF23 (HR per unit SD 1.44 [95%CI 1.19–1.74]; p < 0.001), but not sKlotho (HR per unit SD 0.39 [95%CI 0.09–1.79]; p = 0.227), was associated with an increased risk for the combined end point of death, heart transplantation or assist device implantation. Moreover, in tertile-based sex-adjusted analysis, individuals in the first FGF23 tertile were 4.1 times (95%CI 1.42–12.38; p = 0.009) more likely of freedom from the combined end point compared to individuals in the third tertile (Fig. 2A). No relationship was found between sKlotho and freedom from the combined endpoint (Fig. 2B). Finally, uni- and multivariate Cox regression analysis adjusted for age and sex revealed an association between FGF23 and event-free survival independently of established predictors in heart failure, such as NT-proBNP, LV-EF, and eGFR (Table 2).

**Table 2:** Association between FGF23, sKlotho and established predictors and freedom from death, HTx, or assist device implantation during the observation period using uni- and multivariate, sex- and age-adjusted Cox proportional hazards regression analyses.

	Univariate model				Multivariate model			
	Wald	HR	95% CI	P value	Wald	HR	95% CI	P value
lnFGF23 (RU/ml)	18.95	1.998	1.463–2.729	<0.001	4.8	1.714	1.058–2.774	0.028
lnsKlotho (mg/dl)	0.78	0.722	0.352–1.484	0.376	2.17	0.626	0.336–1.167	0.141
lneGFR (ml/min/1.73 m^2^)	25.65	0.078	0.029–0.210	<0.001	0.28	1.640	0.260–10.396	0.598
lnNT-proBNP (ng/l)	21.35	2.412	1.660–3.504	<0.001	6.50	1.727	1.135–2.626	0.011
LV-EF (%)	3.61	1.025	0.999–1.050	0.057	1.11	1.014	0.988–1.042	0.293

eGFR, estimated glomerular filtration rate; NT-proBNP, N-terminal pro-B-type natriuretic peptide; LV-EF, left ventricular ejection fraction.

### Cardiac mRNA expression of Klotho and FGF23 were upregulated in human DCM hearts

**Figure 3:**
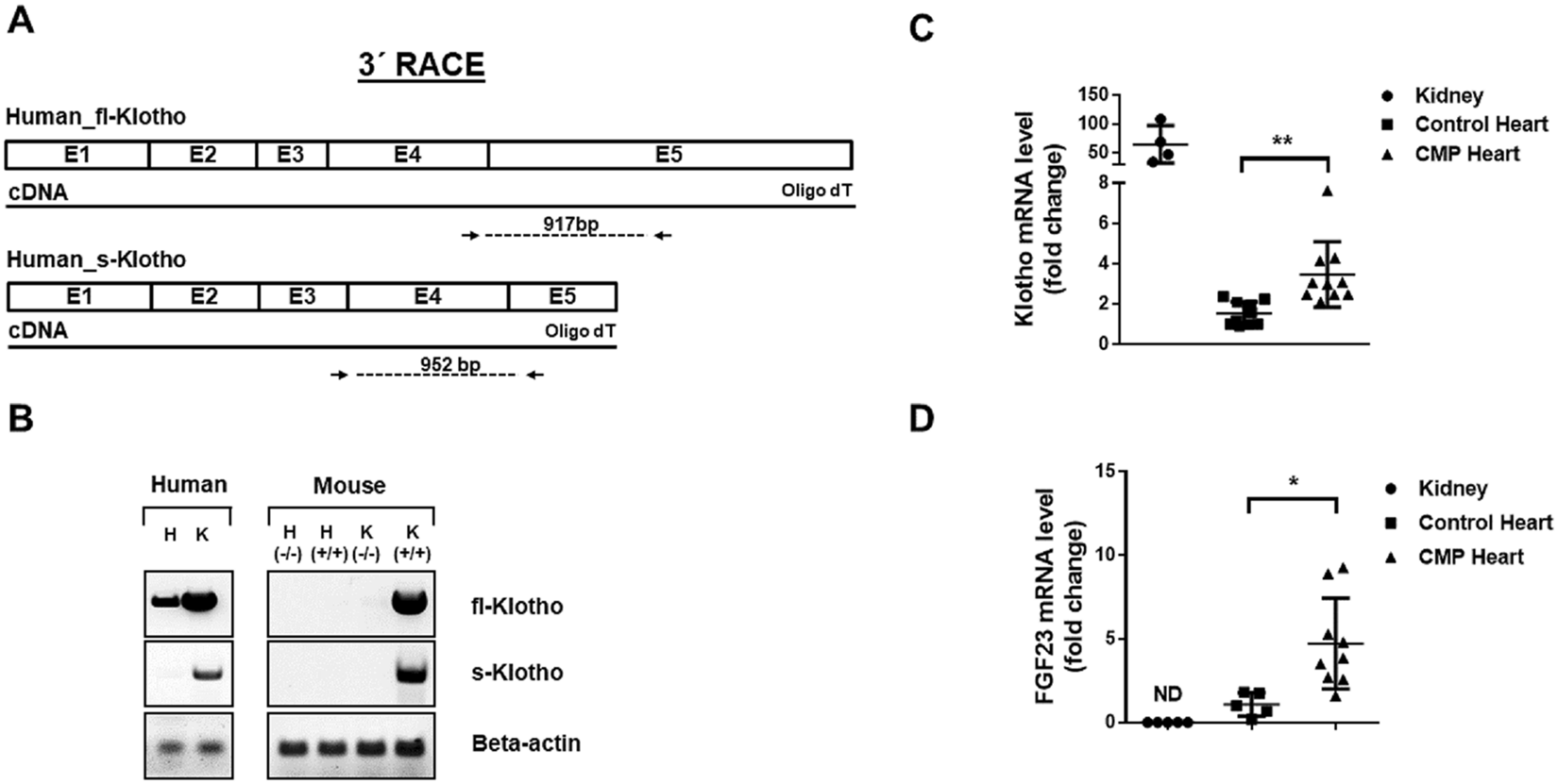
Full-length Klotho mRNA is upregulated in CMP hearts. (**A)** 3′RACE PCR strategy utilizing oligo dT primers to detect the full-length and secreted isoform of Klotho mRNA in the human heart. (**B)** Columns 1–2 (Human Tissue): RT-PCR expression analyses of the full-length (fl-Klotho) and the secreted form (s-Klotho) of Klotho mRNA in human CMP hearts (H) and kidney (K) as positive control. Beta actin was used as internal control. Columns 3–6 (Mouse Tissue): RT-PCR expression analyses of Klotho in KO H (−/−) and WT H (+/+) hearts as well as Klotho KO K (−/−) and WT K (+/+) kidney as controls. The gels were cropped for conciseness. Full length gels are presented in Supplementary Figure S3 and S4. (**C**) Relative amount of Klotho mRNA levels related to the reference gene RPL32 in human kidney (n = 4), control hearts (n = 10) and CMP hearts (n = 10). Shown are all individual data points, lines show mean ± SD. ^**^P ≤ 0.01. (**D**) Relative amount of FGF23 mRNA levels related to the reference gene RPL32 in human kidney (n = 4), controls hearts (n = 10) and CMP hearts (n = 10). Shown are all individual data points, lines show mean ± SD. ^***^P ≤ 0.05. FGF mRNA was not detected (ND) in healthy kidneys.

Since we found no clear relation of sKlotho in the serum to heart failure outcomes we aimed to investigate Klotho expression directly in the human heart. 3′ RACE PCR utilizing oligo dT primers followed by RT-PCR analysis with isoform specific primers revealed expression of full-length Klotho mRNA in human heart and kidney (Fig. 3A,B). In contrast to kidney, we were not able to detect the secreted variant of Klotho (isoform 2) in heart tissue with specific primers recognizing the alternative splice product between exon 3–4. Further sequencing of the Klotho PCR product from human hearts and kidney confirmed the cDNA sequence of full-length Klotho (see Supplementary Figure S1). In contrast to human hearts, we could neither detect full-length nor the secreted isoform of Klotho mRNA in mouse hearts. Quantitative RT-PCR analysis of Klotho mRNA expression in the heart and the kidney as a positive control revealed a significantly upregulation of full-length Klotho in DCM hearts as compared to non-failing control hearts (3.50 ± 0.51 vs. 1.53 ± 0.18; p = 0.002, Fig. 3C). Of note, Klotho mRNA expression levels in the kidney were 100–200 fold higher compared to the heart (Fig. 3C). As shown in Fig. 3D, expression of FGF23 was also significantly upregulated in DCM hearts as compared to controls (4.71 ± 0.90 vs. 1.08 ± 0.31; p ≤ 0.05, respectively), whereas no mRNA expression was detected in healthy kidney tissue. The heart failure marker BNP mRNA was significantly upregulated in DCM hearts as compared to non-failing controls (50.7 ± 10 vs. 4.15 ± 1.63; p ≤ 0.001, see also Supplementary Figure S2).

### Cleaved Klotho rather than the transmembrane protein is upregulated in DCM hearts

**Figure 4:**
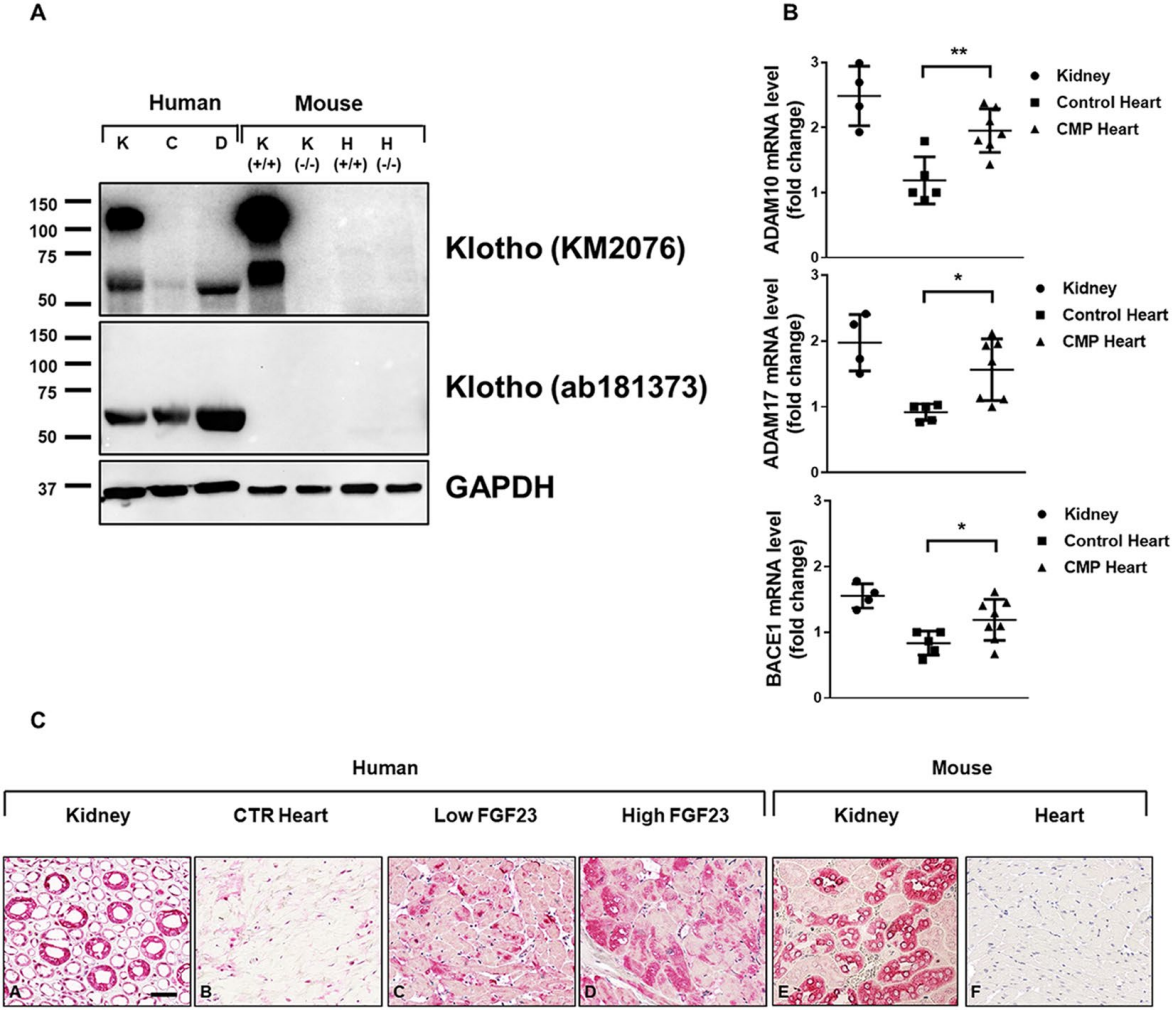
Expression analysis of Klotho protein in kidney and heart tissue. (**A)** Columns 1–3 (Human Tissue): Immunoblot of Klotho and GAPDH in tissue samples derived from kidney (K), non-failing control hearts (**C**), and CMP hearts (**D**). Gel was loaded with 50 µg protein. Note that in the kidney two bands at 130 kDa and 65 kDa are visible, as opposed to only one band at 65 kDa in the heart. (B) Columns 4–7 (Mouse Tissue): Immunoblot of Klotho in KO K (−/−) and WT K (+/+) kidney as controls and KO H (−/−) and WT H (+/+) hearts. Two different antibodies, KM2076 (first row, recognition site: AA 55–261), and ab181373 (second row, recognition site: AA 400–500) with recognition sites in the KL1 region of human Klotho were used. The gels were cropped for conciseness. Full length gels are presented in Supplementary Figure S5. (**B**) Relative amount of ADAM10, ADAM17, and BACE1 mRNA levels related to the reference gene RPL32 in human kidney (n = 4), controls hearts (n = 10) and CMP hearts (n = 10). Shown are all individual data points, lines show mean ± SD. ^***^P ≤ 0.001. (**C)** Human Tissue A-D: Immunohistochemistry staining of Klotho protein in kidney (A), control hearts (B), and CMP hearts from patients with low (C) and high levels (D) of serum FGF23, respectively. Mouse Tissue E-F: Immunohistochemistry staining of Klotho protein in kidney (E), and heart tissue from mice. Scale bar represents 50 µM.

Next, we examined Klotho protein expression in heart tissue derived from CMP and control hearts by Western blot analysis. Tissue samples from kidneys were used as positive controls. As shown in Fig. 4A, immunoblotting of tissue samples from human kidneys revealed two bands corresponding to the transmembrane (130 kDa) and the cleaved form (65 kDa) of sKlotho, whereas in the heart only sKlotho (65 kDa) was detectable utilizing two different antibodies, KM2076 (recognition site: AA 55–261), and ab181373 (recognition site: AA 400–500) with recognition sites in the KL1 domain of human Klotho. sKlotho protein was upregulated in CMP hearts (D) compared to healthy controls (C) (Fig. 4A). The specificity of the KM2076 Ab, which confers known cross-reactivity in mouse tissue, was further confirmed by immunoblotting against kidney and heart tissue derived from WT and Klotho KO mice. Again, we detected two bands in WT mouse tissue from kidney corresponding to the transmembrane and the cleaved form of sKlotho, while no specific Klotho bands were detected in kidney and hearts of Klotho KO mice and in the WT mouse heart. The monoclonal Ab ab181373 is known for its reactivity against human tissue and was not detected in mouse tissue. Further immunohistochemical staining’s with the KM2076 antibody confirmed Klotho protein expression in human kidney samples as controls as well as in human non-failing hearts and CMP tissue from patients with low FGF23 and high FGF23 levels (Fig. 4A–D). Of note, Klotho was upregulated in tissue samples from patients with high FGF23 levels compared to low FGF23 levels. Specific staining was confirmed in mouse kidney tissue, no expression of Klotho was seen the mouse heart.

Since ADAM10, ADAM17 and BACE1 are known proteases involved in the cleavage of Klotho, we analyzed the expression of these enzymes in human heart and kidney. As shown in Fig. 4B, ADAM10, ADAM17 and BACE1 were readily detectable in human hearts as well as in the kidney. The proteolytic enzymes were significantly upregulated in DCM hearts suggesting local cleavage of Klotho in the heart.

## Discussion

In this longitudinal study we demonstrate that FGF23, rather than sKlotho in the serum, appropriately reflects disease severity and progression in CHF. Instead, we show for the first time Klotho mRNA and protein upregulation in human cardiomyopathy, independent of sKlotho levels in the serum suggesting paracrine local affects.

The present analysis confirms the association between FGF23 and disease progression in HF and its potential as a biomarker for risk prediction^8,24,25^. Also, we show a clear dose-response relationship for FGF23 levels with increasing severity of HF. Previous findings from population-based studies demonstrated an association between FGF23 and the advent of incident HF^6,7,26,27^. In addition, circulating FGF23 concentrations have been linked to left ventricular hypertrophy (LVH)^3,5,28^. Corresponding results by Faul *et al*. demonstrated an independent association between elevated FGF23 levels and LVH in a large CKD cohort^5^. Data from the same group indicate hypertrophic growth in isolated neonatal rat cardiomyocytes mediated by FGFR-dependent activation of the calcineurin-NFAT signaling cascade, not requiring Klotho as coreceptor^5,29^.

While Klotho has been studied extensively in experimental models and in patients with CKD, data in human HF are rare. Experimental data in mice suggest that sKlotho exerts cardioprotection through downregulation of the TRPC6 calcium channel^22^. Thereby, sKlotho interrupts a feed-forward loop that amplifies stress-induced abnormal intracellular Ca2+ signaling, which activates the calcineurin-NFAT signaling cascade. In CKD mice Klotho deficiency correlates with the extent of cardiac hypertrophy and fibrosis^30^. Also, in rodents sKlotho protects against uremic cardiomyopathy independently of FGF23 and phosphate^21^. Findings from a clinical study have linked higher Klotho levels to the absence of atrial fibrillation in hemodialysis patients^23^.

In contrast to FGF23, we could not find a clear relationship between sKlotho in the serum and HF severity. Nevertheless, sKlotho levels in our HF cohort were lower by trend than recently reported by a healthy Danish cohort using the same ELISA kit (380 pg/ml vs. 472 pg/ml)^31^. This is in line with findings in patients with renal disease showing significantly lower sKlotho levels than in healthy controls^23^ and decreased Klotho expression early in the course of CKD^32,33^. This phenomenon was explained by the fact that sKlotho derives mainly from renal tubular cells^9^. Findings in our cohort with stable HF do not support a protective effect of circulating sKlotho in heart failure. Although absolute differences in sKlotho levels between patient groups with and without an endpoint were small albeit significant, sKlotho levels were not associated with event-free survival. Corresponding results were recently reported in patients with CKD and ESRD^23,33^.

Instead, we provide for the first-time unambiguous evidence for Klotho mRNA expression in human hearts. Particularly the full-length transcript of Klotho was significantly upregulated in CMP hearts compared to non-failing controls. In contrast to the kidney, we could only detect the full-length trans-membrane form of Klotho mRNA in the heart, whereas the secreted form (isoform 2) was not detectable. Previous reports indicating no relevant expression of Klotho in cardiomyocytes might be explained by the fact that Klotho expression was studied mainly in mice and rodent cell lines, whereas data regarding Klotho expression in the human heart is very limited^5,9,13^. Accordingly, we also failed to detect expression of Klotho in mouse hearts indicating species-specific differences. Using different primer pairs and sequencing of 3´RACE generated cDNA products clearly showed that Klotho mRNA indeed is expressed in the human heart. Klotho amplification in human hearts may have been overlooked in earlier studies since expression of mRNA occurred only after 26 PCR cycles and other primers were used. Based on our experiments we conclude that full-length but not the secreted from of Klotho is expressed in the human heart. Our results were further confirmed by immunoblotting, and immunohistochemistry. Immunoblotting with two different Abs recognizing binding sites in the KL1 region of Klotho revealed a single 65 kDa band corresponding most likely to the cleaved form of sKlotho in the heart^34^. The assumption that upregulated Klotho protein in diseased hearts is derived from cleavage of the membrane-bound Klotho is further supported by our findings that (i) circulating sKlotho levels in serum were neither upregulated nor associated with disease progression in CHF and that (ii) full-length Klotho mRNA was upregulated in diseased hearts, whereas mRNA of secreted Klotho (isoform 2) was not detectable. Furthermore, a recently published study showed that the secreted form of Klotho mRNA (isoform 2) contains premature termination codons priming mRNAs for degradation by nonsense-mediated mRNA decay thereby abolishing protein translation^35^. Moreover, we detected the expression of proteases like ADAM10, ADAM17 and BACE1 capable for cleavage of Klotho at the transmembrane site (alpha-cut) and between the domains KL1 and KL2 (beta-cut)^34^. The fact that we could only detect the 65kDA protein, suggests complete cleavage of transmembrane Klotho into KL1 and KL2 domains in the heart by proteolysis. Interestingly, production of Klotho is 200fold higher in the kidney, but expression of proteolytic enzymes is on the same level as compared to heart tissue. Incomplete cleavage might explain the presence of two bands (130 kDa and 65 kDa) in the kidney compared to the heart. Therefore, our results support the hypothesis that Klotho expression in the heart is independently regulated to serum levels and might be controlled in a local paracrine manner.

Elevated levels of circulating FGF23 are in line with previous findings demonstrating its role in cardiac remodeling and disease progression. Our results support the notion that FGF23 is upregulated in diseased human CMP hearts and might contribute to increased serum levels in chronic heart failure even prior to detoriation of renal function^36,37^. Low circulating Klotho levels in HF and high expression of Klotho in cardiomyopathy, however, are difficult to reconcile. In rodents sKlotho ameliorates cardiac hypertrophy by inhibiting TRPC6 currents in cardiomyocytes^21^. This mechanism is mediated by the insulin-like growth factor 1 receptor and is independent of FGFR and the Klotho receptor. Furthermore, sKlotho protects from isoproterenol induced cardiomyocyte apoptosis via inhibition of p38 and c-Jun NH2-terminal kinase pathways suggesting ameliorating stress induced cardiac damage^38^. A recently published study suggests that sialogangliosides in lipid rafts can act as membrane receptors for sKlotho and that particularly the KL1 domain is sufficient to inhibit raft-dependent PI3K signaling and TRPC6 function thereby protecting against stress-induced cardiac hypertrophy in mice^39^. In fact, the antibodies used in our experiments mainly recognize the KL1 domain (65 kDa) of sKlotho. From this we hypothesize that local overexpression of sKlotho may compensate for Klotho-independent maladaptive effects of FGF23 in an autocrine or paracrine manner in human heart failure.

### Limitations

Data in this study are derived from a single-center cohort of patients with non-ischemic cardiomyopathy. Hence, applicability of findings to patients with ischemic cardiomyopathy is limited. Owing to its nature as a cohort study, our trial lacks a control group that would allow for unequivocal comparison of sKlotho levels. Furthermore, the trigger of local Klotho overexpression remains unresolved and needs to be clarified in further experimental models.

## Conclusions

In this study we show that FGF23 rather than sKlotho in the serum is associated with disease severity and progression of chronic heart failure. Instead, Klotho is upregulated locally in CMP hearts, suggesting paracrine effects.

## Methods

An expanded Methods section on real-time RT-PCR, western blotting, and immunoperoxidase staining is provided in the Supplementary Material. A complete list of amplification primers used in the study is provided in Supplementary Table S1.

### Study population

For this retrospective analysis 287 Caucasian patients with chronic heart failure due to non-ischemic cardiomyopathy and reduced LV-EF, who were referred for further invasive diagnostic evaluation between January 2007 and September 2014 were consecutively included. Inclusion criteria were patients ≥18 years of age with confirmed diagnosis of CHF based on the presence of current or previous symptoms or characteristic clinical signs and evidence of left ventricular dysfunction and normal or mild reduced kidney function. Exclusion criteria were patients with acute HF, vitamin D and/or calcimimetic therapy for the last six months, coronary artery disease (CAD) on coronary angiography or moderate-severe CKD to obviate potential interferences. Patients were treated according to prevailing CHF guidelines. Patients were followed until December 2014 (time of data censoring) or to the occurrence of death, heart transplantation or ventricular assist device (VAD) implantation, which constituted the combined endpoint. Death events were retrieved from the local mortality registry and from the patients’ relatives.

### Tissue samples

Ventricular tissue samples were either obtained by endomyocardial biopsy (EMB) or as dispensable tissue during heart surgery from patients with non-ischemic cardiomyopathy (n = 10) or healthy donor hearts (n = 10) before transplantation. Samples were immediately immersed in RNAlater (Qiagen GmbH) and stored at 4 °C for 24 hours (RNA later incubation time) followed by long-term storage at −80 °C for further RNA processing. Samples were analyzed for the expression of Klotho and BNP mRNA. Healthy kidney tissue (n = 4) was obtained from non-diseased parts of tumor nephrectomy samples. Mouse wild type (WT) and Klotho knock-out (KO) tissues from kidney and heart were kindly provided by Dr. Leifheit-Nestler lab (Hannover, Germany) and Dr. Jakob Völkl (Berlin, Germany).

The study confirmed with the principles outlined in the Declaration of Helsinki and was approved by the local ethics committee of the Medical University of Innsbruck. All patients gave written informed consent for participation in the study.

### Measurements

Fasting blood samples were drawn in all patients at study entry and stored at −80°. All laboratory variables were measured at the central laboratory of Innsbruck Medical University Hospital that undergoes regular internal and external quality audits. FGF23 was determined using an FGF23 immunoassay (Immutopics Inc., San Clemente, CA, USA; interassay coefficient of variation <5%) with polyclonal antibodies detecting both the intact molecule and large carboxyl terminal fragments of human FGF-23. Circulating concentrations of FGF23 are expressed as relative units per milliliter (RU/ml).

Soluble α-Klotho was measured with a solid-phase sandwich ELISA (IBL Co. Ltd., Minneapolis, MN, USA; interassay coefficient of variation <7%). This assay detects the tertiary protein structure of an extracellular domain of KL1 with monoclonal antibodies. Circulating concentrations of soluble α-Klotho are expressed as pg/ml.

### Specimens

Freshly isolated tissues from human CMP, non-failing hearts and kidneys were immediately fixed in 10% neutral buffered formalin for further processing. An expanded Methods section on real-time RT-PCR, western blotting, and immunoperoxidase staining is provided in the supplement.

### Statistical analysis

Data are presented as median (25th, 75th percentile) or n (%), as appropriate. The Shapiro-Wilks test was used to test for normality in distribution of included parameters. We used the Kruskal-Wallis and Pearson Chi-square tests or one-way ANOVA to test for the differences in categorical or continuous factors, respectively, between various categories of FGF23 and sKlotho. Spearman rank correlation was used to assess cross-sectional relations between FGF23, sKlotho and 25 hydroxyvitamin D (25(OH)D). Assessment of the prognostic relevance of FGF23 and sKlotho per standard deviation each for the combined endpoint of freedom from death, heart transplantation or assist device implantation was performed using sex- and age-adjusted Cox proportional hazards regression analysis. Kaplan-Meier estimates and the log-rank test were used to calculate freedom from death, heart transplantation or assist device implantation. A multivariate model for FGF23 and sKlotho per log unit each was calculated with eGFR, NT-proBNP, and LV-EF as co-variates. Differences in mRNA expression by quantitative PCR between biopsies obtained from kidney, diseased hearts and healthy controls were analyzed by one-way ANOVA for multiple group comparisons. P values < 0.05 were considered to indicate statistical significance. Statistical analysis was performed using SPSS 20.0 (SPSS Corp.).

### Data Availability

All data generated or analysed during this study are included in this published article and its Supplementary Information files.

## Electronic supplementary material

Supplementary Material
