# Supplementary material for: Klotho is upregulated in human cardiomyopathy independently of circulating Klotho levels
Source: Sci Rep. 2018 May 30;8:8429. doi: 10.1038/s41598-018-26539-6 (PMC5976633; doi:10.1038/s41598-018-26539-6)
Supplement: Supplementary file 1 — Supplementary Material [file 41598_2018_26539_MOESM1_ESM.doc]

**Supplementary material**

**Klotho is upregulated in human cardiomyopathy independently of circulating Klotho levels**

Poelzl G, MD, Assoc. Prof.^1^**^+^**, Ghadge SK, PhD^1^**^+^**, Messner M, MD^1^, Haubner B, MD^1^, Wuertinger Ph, MD^2^, Griesmacher A, MD, Prof.^2^, Doerler J, MD^1^, Ensinger C, MD, Prof.^3^, Ulmer H, PhD, Prof.^4^, Zaruba MM, MD, Ass. Prof.^1^**^*^**

^1^ Clinical Division of Cardiology and Angiology, Medical University of Innsbruck, Austria

^2^Central Institute for Medical and Chemical Laboratory Diagnosis, Innsbruck Medical University Hospital, Austria

^3^ Department of Pathology, Medical University of Innsbruck, Austria

^4^ Department of Medical Statistics, Informatics and Health Economics, Medical University of Innsbruck, Austria

**^+^**Poelzl G and Ghadge SK contributed equally to this manuscript

^*^Corresponding author: Ass.-Prof. PD Dr. Marc-Michael Zaruba

**Detailed method section**

**3´RACE and qRT-PCR**

Total RNA was extracted from human and mouse heart and kidney tissues samples with Trizol (Invitrogen). 3´RACE PCR was performed to amplify cDNA products utilizing oligo dT primers using the 5′/3′ RACE Kit (Roche) and reverse-transcribed according to the manufacturer's protocol. Gene specific primers for human and mouse full length and secreted Klotho isoforms were designed and amplified using expand long template PCR system (Roche) and PCR products were verified on agarose gels and sequenced. Amplification primers were listed in Supplementary table 1. For quantitative RT-PCR analysis total RNA was reverse-transcribed using the QuantiTect RT kit (Qiagen) according to the manufacturer's protocol. Exon spanning primers for human Klotho, BNP, ADAM10, ADAM17, BACE1 were designed and verified on agarose gels (Supplementary table 1). Cycling conditions for qPCR were 95°C for 10 min (activation), 95°C for 15sec (denaturation) and 60°C for 1min (annealing and extension) up to 40 cycles. Using 2x SYBR green mastermix (Applied Biosystems, Foster City, CA, USA) semiquantitative gene expression was calculated using the comparative ΔΔCt method with RPL32 as a reference gene.

**Western blot**

Tissue samples were snap-frozen in liquid nitrogen and stored at -80°C for later use. Samples were homogenized in RIPA lysis buffer (Thermo Scientific) adding Halt™ protease inhibitor cocktail (Thermo Scientific). After centrifuging for 20 min at 12,000 rpm at 4°C, the protein concentration was analyzed with a Pierce™ BCA protein assay kit (Thermo Scientific). A protein amount of 50 µg for eych sample was loaded and run together on a 4-20% SDS polyacrylamide gel. After transfer to nitrocellulose membrane, the membrane was treated with 5% blocking buffer (5% wt/vol non-fat milk in TBS (20 mM Tris-HCl, pH 7.4, 150 mM NaCl) with 0.1% Tween 20 for 2 hrs at room temperature. After washing, the blots were incubated with antibodies against human Klotho (KM2076, Biologo; Abcam, ab181373) in 1:100 and 1:500 dilution for overnight at 4°C. The membranes were stripped with Restore™ western blot stripping buffer and incubated with a mouse monoclonal antibody against GAPDH (Abcam, ab8245) in a 1:10000 dilution for 60 min at room temperature. Appropriate secondary antibodies conjugated with Horseradish-Peroxidase (1:5000) (GE Healthcare) were incubated for 60 min at RT. Blots were visualized utilizing ECL™ Prime Detection Reagent (GE Healthcare) and ChemicDoc MP Imaging system (Biorad).

**Immunoperoxidase staining**

Deparaffinized sections were blocked with methanol / hydrogen peroxide and boiled at 121°C and 1 bar. Subsequently, sections were incubated with an antibody against human Klotho (KM2076, Biologo) followed by Vectastain Elite ABC kit (PK-6102, Vector laboratories, Cambridgeshire, UK) and Vector® Red substrate kit (SK-5100, Vector Laboratories, Cambridgeshire, UK) staining.

**Supplemetary Figure S1. Alignment of human PCR product cDNA sequence to full-length human Klotho mRNA**

3´RACE generated cDNA products derived from human Kidney (3´RACE_Kidney) and human heart tissue (3´RACE_Heart) were aligned to the known Klotho mRNA sequence (NM_004795.3) demonstrating 100% alignment with the known sequence of full-length human Klotho.

3´RACE_Kidney ----------------GCGGTATGGTATCAATCTTTGCGGATACTTTGCTTATTCGTTTA 44

NM_004795.3 CTCTCAAAGCCCACATACTGGATGGTATCAATCTTTGCGGATACTTTGCTTATTCGTTTA 2760

3´RACE_Heart -------------CATACTGGATGGTATCAATCTTTGCGGATACTTTGCTTATTCGTTTA 47

3´RACE_Kidney ACGACCGCACAGCTCCGAGGTTTGGCCTCTATCGTTATGCTGCAGATCAGTTTGAGCCCA 104

NM_004795.3 ACGACCGCACAGCTCCGAGGTTTGGCCTCTATCGTTATGCTGCAGATCAGTTTGAGCCCA 2820

3´RACE_Heart ACGACCGCACAGCTCCGAGGTTTGGCCTCTATCGTTATGCTGCAGATCAGTTTGAGCCCA 107

3´RACE_Kidney AGGCATCCATGAAACATTACAGGAAAATTATTGACAGCAATGGTTTCCCGGGCCCAGAAA 164

NM_004795.3 AGGCATCCATGAAACATTACAGGAAAATTATTGACAGCAATGGTTTCCCGGGCCCAGAAA 2880

3´RACE_Heart AGGCATCCATGAAACATTACAGGAAAATTATTGACAGCAATGGTTTCCCGGGCCCAGAAA 167

3´RACE_Kidney CTCTGGAAAGATTTTGTCCAGAAGAATTCACCGTGTGTACTGAGTGCAGTTTTTTTCACA 224

NM_004795.3 CTCTGGAAAGATTTTGTCCAGAAGAATTCACCGTGTGTACTGAGTGCAGTTTTTTTCACA 2940

3´RACE_Heart CTCTGGAAAGATTTTGTCCAGAAGAATTCACCGTGTGTACTGAGTGCAGTTTTTTTCACA 227

3´RACE_Kidney CCCGAAAGTCTTTACTGGCTTTCATAGCTTTTCTATTTTTTGCTTCTATTATTTCTCTCT 284

NM_004795.3 CCCGAAAGTCTTTACTGGCTTTCATAGCTTTTCTATTTTTTGCTTCTATTATTTCTCTCT 3000

3´RACE_Heart CCCGAAAGTCTTTACTGGCTTTCATAGCTTTTCTATTTTTTGCTTCTATTATTTCTCTCT 287

3´RACE_Kidney CCCTTATATTTTACTACTCGAAGAAAGGCAGAAGAAGTTACAAATAGTTCTGAACATTTT 344

NM_004795.3 CCCTTATATTTTACTACTCGAAGAAAGGCAGAAGAAGTTACAAATAGTTCTGAACATTTT 3060

3´RACE_Heart CCCTTATATTTTACTACTCGAAGAAAGGCAGAAGAAGTTACAAATAGTTCTGAACATTTT 347

3´RACE_Kidney TCTATTCATTCATTTTGAAATAATTATGCAGACACATCAGCTGTTAACCATTTGCACCTC 404

NM_004795.3 TCTATTCATTCATTTTGAAATAATTATGCAGACACATCAGCTGTTAACCATTTGCACCTC 3120

3´RACE_Heart TCTATTCATTCATTTTGAAATAATTATGCAGACACATCAGCTGTTAACCATTTGCACCTC 407

3´RACE_Kidney TAAGTGTTGTGAAACTGTAAATTTCATACATTTGACTTCTAGAAAACATTTTTGTGGCTT 464

NM_004795.3 TAAGTGTTGTGAAACTGTAAATTTCATACATTTGACTTCTAGAAAACATTTTTGTGGCTT 3180

3´RACE_Heart TAAGTGTTGTGAAACTGTAAATTTCATACATTTGACTTCTAGAAAACATTTTTGTGGCTT 467

3´RACE_Kidney ATGACAGAGGTTTTGAAATGGGCATAGGTGATCGTAAAATATTGAATAATGCGAATAGTG 524

NM_004795.3 ATGACAGAGGTTTTGAAATGGGCATAGGTGATCGTAAAATATTGAATAATGCGAATAGTG 3240

3´RACE_Heart ATGACAGAGGTTTTGAAATGGGCATAGGTGATCGTAAAATATTGAATAATGCGAATAGTG 527

3´RACE_Kidney CCTGAATTTGTTCTCTTTTTGGGTGATTAAAAAACTGACAGGCACTATAATTTCTGTAAC 584

NM_004795.3 CCTGAATTTGTTCTCTTTTTGGGTGATTAAAAAACTGACAGGCACTATAATTTCTGTAAC 3300

3´RACE_Heart CCTGAATTTGTTCTCTTTTTGGGTGATTAAAAAACTGACAGGCACTATAATTTCTGTAAC 587

3´RACE_Kidney ACACTAACAAAAGCATGAAAAATAGGAACCACACCAATGCAACATTTGTGCAGAAATTTG 644

NM_004795.3 ACACTAACAAAAGCATGAAAAATAGGAACCACACCAATGCAACATTTGTGCAGAAATTTG 3360

3´RACE_Heart ACACTAACAAAAGCATGAAAAATAGGAACCACACCAATGCAACATTTGTGCAGAAATTTG 647

3´RACE_Kidney AATGACAAGATTAGGAATATTTTCTTCTGCACCCACTTCTAAATTTAATGTTTTTCTGGA 704

NM_004795.3 AATGACAAGATTAGGAATATTTTCTTCTGCACCCACTTCTAAATTTAATGTTTTTCTGGA 3420

3´RACE_Heart AATGACAAGATTAGGAATATTTTCTTCTGCACCCACTTCTAAATTTAATGTTTTTCTGGA 707

3´RACE_Kidney AGTAGTAATTGCAAGAGTTCGAATAGAAAGTTATGTACCAAGTAACCATTTCTCAGCTGC 764

NM_004795.3 AGTAGTAATTGCAAGAGTTCGAATAGAAAGTTATGTACCAAGTAACCATTTCTCAGCTGC 3480

3´RACE_Heart AGTAGTAATTGCAAGAGTTCGAATAGAAAGTTATGTACCAAGTAACCATTTCTCAGCTGC 767

3´RACE_Kidney CATAATAATGCCTAGTGGCTTCCCCTCTGTCAAATCTAGTTTCCTATGGAAAAGAAGATG 824

NM_004795.3 CATAATAATGCCTAGTGGCTTCCCCTCTGTCAAATCTAGTTTCCTATGGAAAAGAAGATG 3540

3´RACE_Heart CATAATAATGCCTAGTGGCTTCCCCTCTGTCAAATCTAGTTTCCTATGGAAAAGAAGATG 827

3´RACE_Kidney GCAGATACAGGAGAGACGCGA--------------------------------------- 845

NM_004795.3 GCAGATACAGGAGAGACGACAGAGGGTCCTAGGCTGGAATGTTCCTTTCGAAAGCAATGC 3600

3´RACE_Heart GCAGATACAGGAGAACACAGG--------------------------------------- 848

**Supplementary Figure S2. BNP mRNA is upregulated in CMP hearts**

Relative amount of BNP mRNA levels related to the reference gene RPL32 in human kidney (n=4), controls hearts (n=10) and CMP hearts (n=10). Shown are all individual data points, lines show mean ± SD. *** P ≤ 0.001. BNP mRNA was not detected (ND) in healthy kidneys.

**
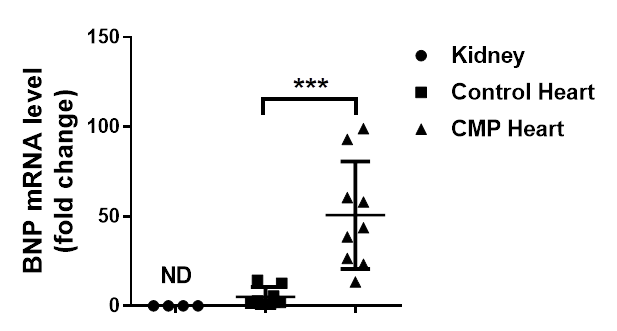
**

**Supplementary Figure S3**

Human

**
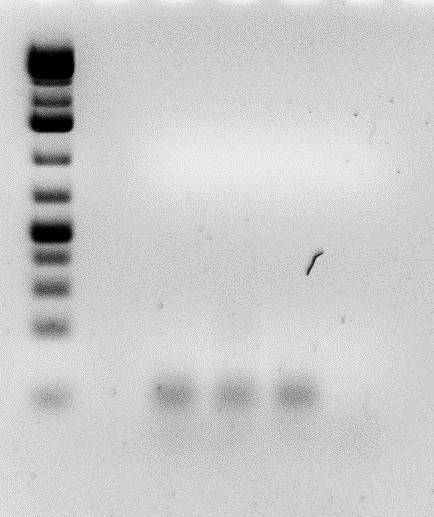

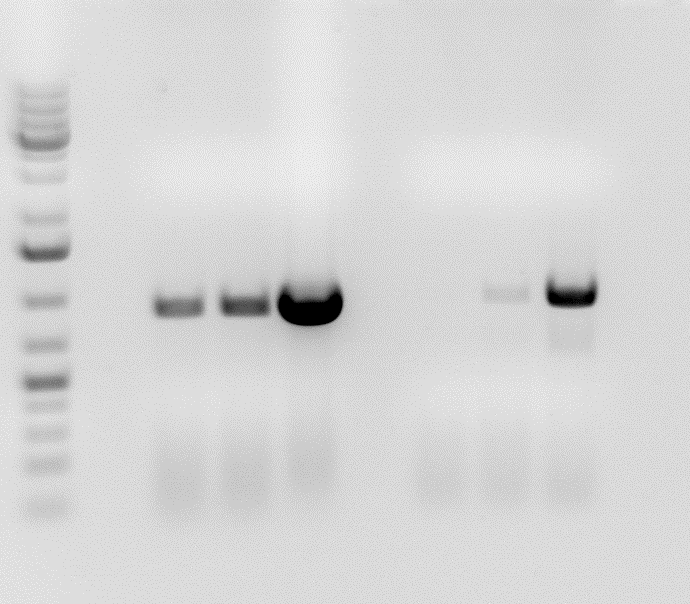
**

H

H

K

H

H

K

H

H

K

**fl-klotho**

**s-klotho**

**Beta-actin**

**500 bp**

**400 bp**

**300 bp**

**200 bp**

**75 bp**

**700 bp**

**1000 bp**

**1500 bp**

**500 bp**

**400 bp**

**300 bp**

**200 bp**

**75 bp**

**700 bp**

**1000 bp**

**1500 bp**

**Supplementary Figure S4**

**
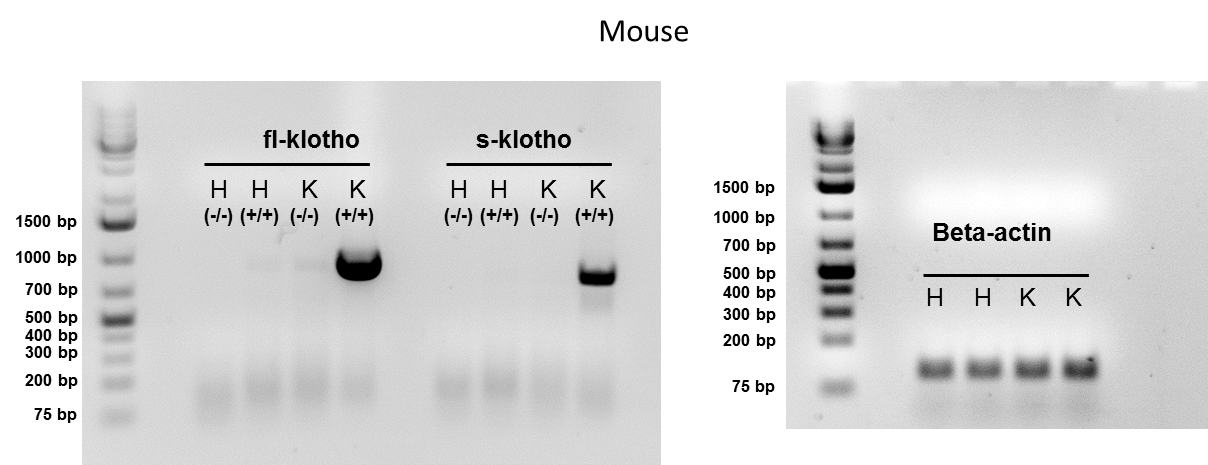
**

**Supplementary Figure S5**

**
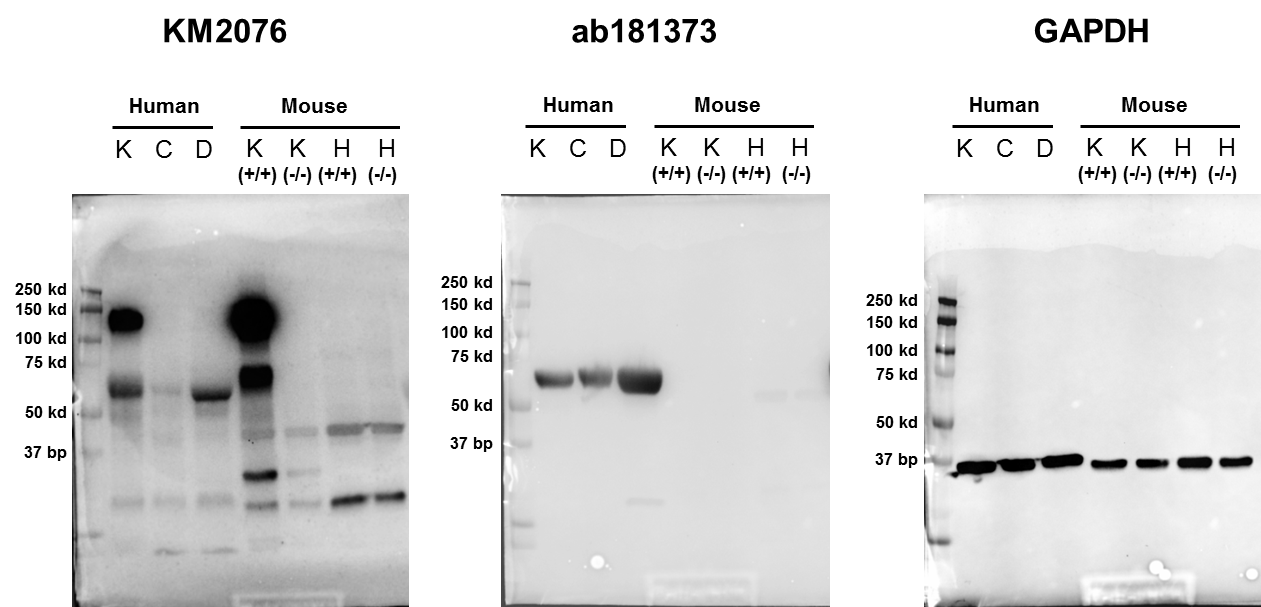
**

**Supplementary Table S1. Primer sequences**

| **Gene** | **Forward primer** | **Reverse primer** |
| --- | --- | --- |
| **H_fl-KLOTHO (3´RACE)** | 5´ CGACCAGCTGAGGGTGTATT 3´ | 5´ CCTAGGACCCTCTGTCGTCT 3´ |
| **H_s-KLOTHO (3´RACE)** | 5´ ACCAATCAGCAGTCTCACCAA 3´ | 5´ GAGTTGAGCCACGTGATGTC 3´ |
| **H_BACT** | 5´ GCCGATCCACACGGAGTACT 3´ | 5´ CTGGCACCCAGCACAATG 3´ |
| **M_fl-KLOTHO (3´RACE)** | 5´ TGGAATCGATGATGACCCCC 3´ | 5´ CTTAATGGGCTGCTCTGGACT 3´ |
| **M_s-KLOTHO (3´RACE)** | 5´ GAGGCAGCTTCTGTCTTGGATA 3´ | 5´ CTGGAGTGGGGCATTTACAGT 3´ |
| **M_BACT** | 5´ CGCCACCAGTTCGCCATGGA 3´ | 5´ TACAGCCCGGGGAGCATCGT 3´ |
| **H_KLOTHO** | 5´ ACCAAGAGAGATGATGCCAAATA 3´ | 5´ CCACTCGAAACCATCCATGA 3´ |
| **H_BNP** | 5´ TCAGCCTCGGACTTGGAAAC 3´ | 5´ TCTTAATGCCGCCTCAGCAC 3´ |
| **H_ADAM10** | 5´ AGACTCGTGGTGGCACATTT 3´ | 5´ TGAATGATCTGCACAGCCCC 3´ |
| **H_ADAM17** | 5´ CCATAGCTGTGAGTGGCGAT 3´ | 5´ CACCCTCGAGTTCCCACAAA 3´ |
| **H_BACE1** | 5´ GCAGGGCTACTACGTGGAGA 3´ | 5´ GTATCCACCAGGATGTTGAGC 3´ |
| **H_RPL32** | 5´AGTTCCTGGTCCACAACGTC 3´ | 5´-CTCTTTCCACGATGGCTTTG-3´ |

**H=Human, M=mouse, fl=full-length, s=secreted**

**Supplementary Table 2. Patient characteristics**

| Variable | Total cohort | | sKlotho  (≤380 pg/ml) | | sKlotho  (> 380 pg/ml) | |  |
| --- | --- | --- | --- | --- | --- | --- | --- |
|  | n = 287 | | n = 145 | | n = 142 | |  |
|  | Median or % | IQR | Median or % | IQR | Median or % | IQR | P |
| Demographic and clinical characteristics |  |  |  |  |  |  |  |
| Age (years) | 48 | 38 - 58 | 50 | 40 - 60 | 48 | 36 - 56 | 0.159 |
| Gender (male) | 68% |  | 70.2% |  | 66.7% |  | 0.608 |
| LV-EF (%) Ventr | 32 | 21 – 46 | 31 | 20 - 45 | 33 | 23 - 48 | 0.366 |
| Heart rate (bpm) | 71 | 61 – 83 | 71 | 62 - 82 | 70 | 66 - 84 | 0.697 |
| Syst. BP (mmHg) | 120 | 110 – 135 | 120 | 110 - 135 | 120 | 110 - 135 | 0.363 |
| BMI | 25.5 | 22.7 – 28.4 | 25.6 | 22.9 - 28.8 | 25.3 | 22.1 – 27.9 | 0.240 |
| NYHA Class |  |  |  |  |  |  | 0.074 |
| NYHA Class I | 24.1% |  | 23.6% |  | 25.2% |  |  |
| NYHA Class II | 44.4% |  | 47.1% |  | 34.5% |  |  |
| NYHA Class III/IV | 23.5% |  | 29.3% |  | 40.3% |  |  |
| Duration of heart failure (months) | 2 | 1 – 8 | 2 | 1 – 11 | 2 | 1 – 6 | 0.290 |
| Hypertension | 42% |  | 40.9% |  | 45.7% |  | 0.466 |
| A-Fib | 11% |  | 10.7% |  | 10.9% |  | 1.0 |
| **Laboratory testing (serum)** |  |  |  |  |  |  |  |
| Ct-FGF23 | 22.6 | 13.1 – 47.6 | 21.8 | 11.7 – 47.9 | 22.9 | 14.4 – 47.0 | 0.472 |
|  |  |  |  |  |  |  |  |
| NT-proBNP (ng/l) | 1191 | 437 – 3147 | 1392 | 447 - 3076 | 1053 | 383 - 3193 | 0.583 |
| eGFR (ml/min/1.73 m^2^) | 75 | 61 - 92 | 73 | 58 - 92 | 77 | 65 - 94 | 0.221  1 |
| P_i_ (mg/dl) | 3.4 | 3.0 – 3.8 | 3.4 | 3.0 – 3.8 | 3.4 | 3.0 – 3.9 | 0.429 |
| PTH (ng/l) 173 | 33.6 | 25.7 – 49.6 | 34.7 | 27.6 – 49.1 | 32.3 | 24.0 – 49.9 | 0.741 |
| 25(OH)D, nmol/l 181 | 44 | 27.9 – 65.5 | 40.8 | 26.2 – 63.0 | 49.8 | 34.3 – 67.0 | 0.073 |
| **Hemodynamics** |  |  |  |  |  |  |  |
| CVP, mmHg | 9 | 6 – 13 | 9 | 7 – 13 | 9 | 6 – 12 | 0.217 |
| Mean PAP, mmHg | 27 | 19 – 34 | 28 | 19 – 36 | 24 | 19 – 32 | 0.127 |
| PCWP, mmHg | 17 | 11 – 25 | 17 | 11 – 25 | 16 | 11 – 24 | 0.514 |
| CI, l/min/qm | 2.0 | 1,7 – 2.5 | 2.0 | 1.7 – 2.4 | 2.1 | 1.7 – 2.7 | 0.188 |
| **Medication** |  |  |  |  |  |  |  |
| ACE inhibitor/ARB | 78.4% |  | 81.4% |  | 77.0% |  | 0.379 |
| Beta-blocker | 76.6% |  | 76.4% |  | 78.4% |  | 0.583 |
| MRA | 37.9% |  | 38.6% |  | 48.1% |  | 1.0 |
| Diuretics | 56.4% |  | 62-1% |  | 51.8% |  | 0.116 |
| Cardiac glycosides | 5.0% |  | 7.1% |  | 2.9% |  | 0.163 |

Data from 282 patients are reported as median (interquartile range) or number (percentage) related to low and high sKlotho plasma levels.

LV-EF, left ventricular ejection fraction; Syst. BP, systolic blood pressure; BMI, Body Mass Index; A-Fib, atrial fibrillation; NT-proBNP, N-terminal pro-B-type natriuretic peptide; eGFR, estimated glomerular filtration rate; PTH, parathormone; Pi, serum phosphate; 25(OH)D, 25-hydroxyvitamin D; CVP, central venous pressure; mean PAP, mean pulmonary artery pressure; PCWP, pulmonary capillary wedge pressure; CI, cardiac index; ACE inhibitor/ARB, angiotensin-converting enzyme inhibitor / angiotensin receptor blocker; MRA, mineralocorticoid receptor antagonist.
